# Role of microRNAs as Clinical Cancer Biomarkers for Ovarian Cancer: A Short Overview

**DOI:** 10.3390/cells9010169

**Published:** 2020-01-09

**Authors:** Cristina Elena Staicu, Dragoș-Valentin Predescu, Călin Mircea Rusu, Beatrice Mihaela Radu, Dragos Cretoiu, Nicolae Suciu, Sanda Maria Crețoiu, Silviu-Cristian Voinea

**Affiliations:** 1 Department of Anatomy, Animal Physiology and Biophysics, Faculty of Biology, University of Bucharest, 91–95 Splaiul Independenței, 050095 Bucharest, Romania; elena.necsulescu@drd.unibuc.ro (C.E.S.); calin.rusu@nipne.ro (C.M.R.); beatrice.radu@bio.unibuc.ro (B.M.R.); 2 Center for Advanced Laser Technologies (CETAL), National Institute for Laser, Plasma and Radiation Physics, 409 Atomiștilor St., 77125 Măgurele, Romania; 3 Department of General Surgery, Sf. Maria Clinical Hospital, ‘Carol Davila’ University of Medicine and Pharmacy, 37–39 Ion Mihalache Blvd., 011172 Bucharest, Romania; drpredescu@yahoo.com; 4 Department of Life and Environmental Sciences, ‘Horia Hulubei’ National Institute of Physics and Nuclear Engineering, 30 Reactorului, 077125 Măgurele, Romania; 5 Life, Environmental and Earth Sciences Division, Research Institute of the University of Bucharest (ICUB), University of Bucharest, 91–95 Splaiul Independenţei, 050095 Bucharest, Romania; 6 Department of Cell and Molecular Biology and Histology, Carol Davila University of Medicine and Pharmacy, 8 Eroii Sanitari Blvd., 050474 Bucharest, Romania; dragos@cretoiu.ro; 7 Fetal Medicine Excellence Research Center, ‘Alessandrescu-Rusescu’ National Institute of Mother and Child Health, 120 Lacul Tei Blvd., 020395 Bucharest, Romania; nsuciu54@yahoo.com; 8 Department of Surgical Oncology, Alexandru Trestioreanu Oncology Institute, ‘Carol Davila’ University of Medicine and Pharmacy, 252 Fundeni Rd., 022328 Bucharest, Romania; dr.voineasilviu@gmail.com

**Keywords:** ovarian cancer, microRNA, biomarker, early diagnosis, molecular clustering analysis

## Abstract

Ovarian cancer has the highest mortality rate among gynecological cancers. Early clinical signs are missing and there is an urgent need to establish early diagnosis biomarkers. MicroRNAs are promising biomarkers in this respect. In this paper, we review the most recent advances regarding the alterations of microRNAs in ovarian cancer. We have briefly described the contribution of miRNAs in the mechanisms of ovarian cancer invasion, metastasis, and chemotherapy sensitivity. We have also summarized the alterations underwent by microRNAs in solid ovarian tumors, in animal models for ovarian cancer, and in various ovarian cancer cell lines as compared to previous reviews that were only focused the circulating microRNAs as biomarkers. In this context, we consider that the biomarker screening should not be limited to circulating microRNAs *per se*, but rather to the simultaneous detection of the same microRNA alteration in solid tumors, in order to understand the differences between the detection of nucleic acids in early vs. late stages of cancer. Moreover, in vitro and in vivo models should also validate these microRNAs, which could be very helpful as preclinical testing platforms for pharmacological and/or molecular genetic approaches targeting microRNAs. The enormous quantity of data produced by preclinical and clinical studies regarding the role of microRNAs that act synergistically in tumorigenesis mechanisms that are associated with ovarian cancer subtypes, should be gathered, integrated, and compared by adequate methods, including molecular clustering. In this respect, molecular clustering analysis should contribute to the discovery of best biomarkers-based microRNAs assays that will enable rapid, efficient, and cost-effective detection of ovarian cancer in early stages. In conclusion, identifying the appropriate microRNAs as clinical biomarkers in ovarian cancer might improve the life quality of patients.

## 1. Introduction

Ovarian cancer occupies a leading position worldwide, and it is considered to be one of the most lethal cancers. In the United States, there are 22,530 new cases estimated for 2019, while the mortality is estimated to be 13,980 deaths in the same year [1]. Ovarian tumors are classified according to the tissue of origin according to the World Health Organization (WHO) classification of ovarian neoplasms, as follows: surface epithelial (65%), germ cell (15%), sex cord-stromal (10%), metastases (5%), and miscellaneous [2]. The most common form of ovarian cancer, epithelial ovarian cancer (EOC), represents more than 90% of malignant tumors and is the fifth leading cause of cancer-related death among women [3]. Recent progress was made in understanding the fundamental biology of this disease. However, this did not influence the mortality rate which has been stagnant since the 80s. It is necessary for a thorough screening based on the presence of biomarkers in conjunction with imaging explorations in order to increase the chances of detection of ovarian cancer in the early stages. Today, ovarian cancer is detected in advanced stages (stage III or IV) at the earliest stage II, based on transvaginal ultrasonography and increasing levels of CA 125 because of the absence of symptomatology [4]. The urgency to find reliable bio screening markers to assure early-stage diagnosis is high.

The definition of a biomarker differs slightly between the National Cancer Institute’s (NCI), which considers that it must be an indicator of an existing process in the body ‘a biological molecule found in blood, other bodily fluids, or tissues that is a sign of a normal or abnormal process, or of a condition or disease’ [5], and that of WHO, which considers it to be an indicator of the evolution of the disease ‘any substance, structure or process that can be measured in the body or its products and influence or predict the incidence of outcome or disease’ [6]. Regardless of the definition, it is clear that an ideal biomarker is a substance or molecule that can be readily detected while using non-invasive methods, which are most likely found in blood or urine. Besides, its presence must provide reliable data on the diagnosis, evolution, and prognosis of the disease. Currently, among the biomarkers that are considered to have potential in the early detection and monitoring of different types of cancer, there are the small, highly conserved non-coding RNA molecules—microRNAs (miRNAs).

**Figure 1:**
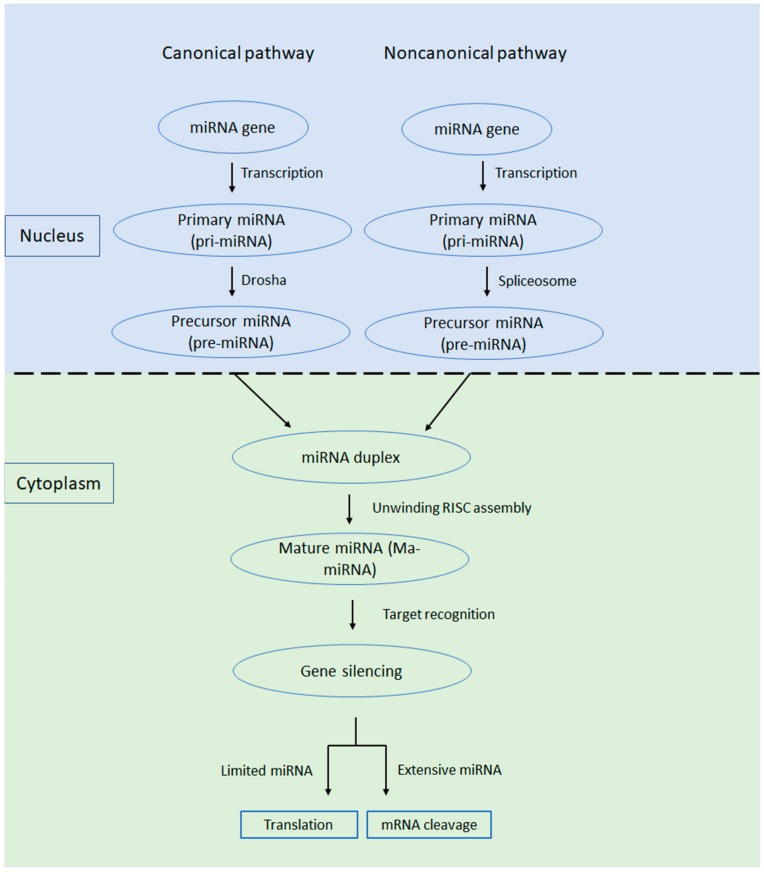
miRNA biogenesis involves five steps: *1. Transcription*. Most miRNAs genes are transcribed by RNA polymerase II. However, a few miRNAs genes use RNA polymerase III. The initial long primary transcript is named primary microRNA (pri-miRNA) and is a hairpin containing transcript with imperfect double-stranded regions. *2. First Cleavage*. The Microprocessor complex formed by DROSHA nuclease and RNA-binding protein DGCR8 removes the 5′ and 3′ ends of the pri-miRNA. The result is a pre-microRNA (pre-miRNA), a short hairpin of about 60 to 70 nt. 3. *Nuclear export*. pre-miRNA is translocated through nuclear pore into the cytoplasm, and after that, it is bound up and form a complex with Exportin-5, Ran, and GTP. *4. Second cleavage*. In the cytoplasm, the pre-miRNA interacts with RNA-Induced Silencing Complex (RISC) loading complex (DICER1, plus an Argonaute protein and either TARBP2 or PRKRA) and is cleaved by DICER1 to a double-stranded miRNA (21 to 23 nucleotides) with a two-nucleotide 3′ overhang of 2-3 nt. *5. Incorporation into RNA-RISC and strand selection*. The double-stranded miRNA is passed to the Argonaute protein where the passenger strand, will be removed and degraded, while the guide strand (the less Table 5′ end), will be selected. RISC can now regulate the gene expression of the mRNA transcript.

miRNAs are involved in the regulation of gene expression in a range of developmental and physiological processes, while their dysregulation has been associated with all types of cancer. The first miRNA, lin-4, was discovered in Caenorhabditis elegans at the beginning of the 1990s [7]. miRNAs are a class of single-stranded, short segments of RNA (of ~22 nt) that can suppress gene expression by binding to complementary segments of messenger RNA and interfere with the formation of proteins by translation [8,9]. In detail, miRNAs interact with target mRNAs and consequently induce mRNA degradation and/or repression of their translation [10]. In most cases, miRNAs interact with the 3′ untranslated region (3′ UTR) of mRNA, but their interaction with other regions was also reported, such as the 5′ UTR, coding sequence, and gene promoters [11]. The miRNAs are synthesized in the nucleus as primary miRNAs (pri-miRNAs), being cleaved by enzymes, like Drosha and Pasha, into precursor miRNAs (pre-miRNAs), and are then transferred into the cytoplasm through the nuclear pores [10,11]. Once in the cytoplasm, pre-miRNAs are cleaved again by Dicer enzymes to form 22 nucleotide long mature miRNAs (Figure 1). Mature miRNAs will become part of the RNA-induced silencing complex (RISC), a multiprotein complex (for more details, see an extensive review by [12].

miRNAs, which typically target tumor-suppressive protein-coding transcripts, are classified as onco-miRs and are found to be upregulated in different types of cancer [13]. On the contrary, miRNAs, which are responsible for the downregulation of oncogenes, are known as tumor suppressor miRNAs and are often lost in cancer [14].

**Figure 2:**
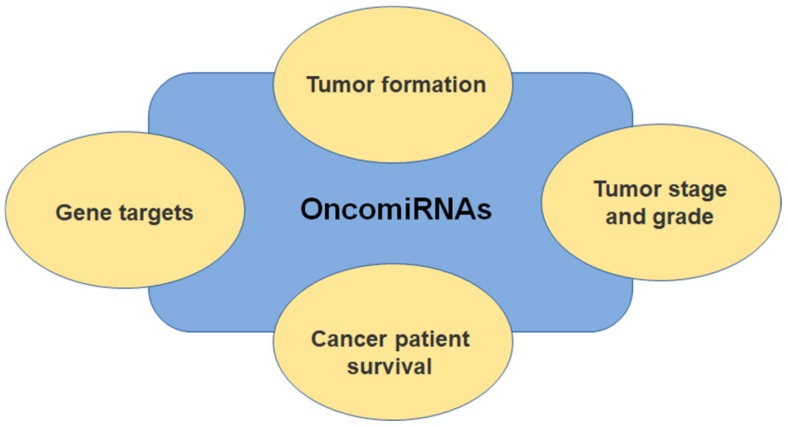
OncomiRNAs shown promise in cancer studies due to their stability and specificity to cells and tumors. Observe their association with gene targeting, tumor formation, cancer patient survival, and tumor stage and grade. OncomiRNAs associations raise the possibility of anticancer therapeutics by using miRNA inhibitors.

In the human genome, a number of approximately 1000 miRNAs have been identified until now by bioinformatic analyses, and it suggested that they contribute to regulating almost 60% of the transcriptome [15]. Nowadays, more and more new genes encoding miRNAs are being described in the literature, with their expression greatly differing from tissue to tissue, which leads to the idea of using miRNAs as biomarkers [16]. Some tumor types have a specific miRNA signature, which, if determined, could be indicative of the existence, progression, and metastasis [17]. Numerous studies that are focused on studying miRNAs expression in cancer showed that some miRNAs behave as tumor-promoting miRNAs (oncomiRNAs and metastamiRNAs), while others are responsible for tumor suppression [18]. Figure 2 aids us in comprehensively understanding the oncomiRNAs association and involvement in etiology, evolution, and prognostic. While taking the oncogenic potential and the tumor-suppressive function of miRNAs into account, it is currently believed that we could detect new strategies for cancer therapy by manipulating miRNAs expression levels. Wong et al. launched *OncomiR* a user-friendly online resource for exploring miRNA dysregulation in cancer and allow flexible miRNomic analysis across many cancer types [19].

The clinical importance of miRNAs has been demonstrated for several types of cancer, including ovarian cancer [20]. The tendency is to determine the extracellular miRNAs that are present in various body fluids, which are stable because they are packed in extracellular vesicles [21].

Several review studies have summarized the potential role of circulating nucleic acids in the diagnostic and prognostic of ovarian cancer [22,23,24]. In detail, Giannopoulou et al. described recent advances on circulating tumor cells and circulating tumor DNA based on liquid biopsy analysis in ovarian cancer and their potential in diagnosis, prognostic and predictive tumor biomarkers [22]. Additionally, it was highlighted the potential of circulating miRNAs in the diagnosis and prognostic of epithelial ovarian carcinoma, with special attention to the subtypes of ovarian cancer, the sample size, the sample subtype (i.e., miRNAs in body fluids), the method of detection, and the survival correlation etc. [23,24]. However, these reviews were limited to the analysis of circulating RNAs/DNAs as biomarkers in ovarian cancer. Our review is focused on the therapeutic potential of miRNAs in ovarian cancer and integrates information regarding circulating miRNAs with their expression changes in ovarian tumors and in ovarian cancer cell lines to offer a new perspective on their quantification in different environments to increase the robustness and reproducibility of using certain miRNAs as reliable biomarkers for the diagnosis/prognosis of this pathology.

## 2. Types of miRNAs in Ovarian Cancer (Circulating Cell-Free and Exosomal miRNAs)

Free circulating miRNAs are considered to be valuable biomarkers in multiple pathologies. After their biogenesis, miRNAs are secreted from cells, and they can be found in a variety of body fluids, such as plasma/serum, saliva, urine, breast milk, cerebrospinal fluid, ascites, pleural effusion, and vaginal discharge [22,23,24,25,26,27,28,29]. Circulating miRNAs can be encountered as cell-free miRNAs that are bound in specific association with protein Argonaute 2 (AGO2) or high-density lipoproteins (HDLs) encapsulated in extracellular vesicles (EVs) of three types: exosomes, microvesicles, and apoptotic bodies [9,30,31,32]. Exosomes contain only a small fraction of circulating miRNAs [33], while larger vesicles (microvesicles or oncosomes) contain the majority of circulating miRNAs, as well as larger RNAs [34].

There is no doubt that circulating cell-free miRNA are considered to be clinically relevant for ovarian cancer diagnosis, prognosis, and therapeutics [35]. However, multiple issues concerning the standardization of miRNA processing, from sample collection and sample storage to RNA isolation, RNA reverse-transcription, and data analyses, should be solved before considering miRNA as reliable biomarkers for current clinical use, despite the great clinical potential of miRNA [36]. Additionally, miRNA are commonly packaged in exosomes, and multiple exosomal miRNAs have been considered as biomarkers in ovarian cancer [37,38,39]. As in the case of miRNA processing, similar concerns exist regarding the standardization of exosomes separation, purification, and analysis [40], which are responsible for hampering the clinical use of exosomal miRNAs in ovarian cancer diagnosis, prognosis, and therapeutics.

The miRNA profile detected in circulating tumor exosomes found in the plasma obtained from ovarian cancer patients was considered to be similar to the miRNA profile in cellular ovarian tumors. Several miRNAs, such as miR-21, miR-141, miR-200a, miR-200b, miR-200c, miR-203, miR-205, and miR-214, were found in the plasma of these patients [41]. miR-30a-5p was detected in exosomes that were extracted from the urine of ovarian serous adenocarcinoma patients and might serve as a promising diagnostic and therapeutic target [42]. Ovarian cancer ascites also contain miR-21, miR-23b, miR-29a, and it is considered that exosomes that are derived from ovarian cancer effusion supernatants are responsible for inducing more aggressive disease [23].

Zhang et al. demonstrated that plasma exosomes from patients with ovarian cancer and healthy women differently expressed miRNAs through high-throughput sequencing: 34 were found to be upregulated and 31 downregulated, respectively. The hsa-miR-106a-5p, hsa-let-7d-5p, and hsa-miR-93-5p expression levels were significantly increased, whereas hsa-miR-122-5p, hsa-miR-185-5p, and hsa-miR-99b-5p were significantly decreased in patients with ovarian cancer comparative with healthy controls [43]. Another study that deals with circulating miRNA profiling in plasma samples of ovarian cancer patients detected a variety of differentially expressed miRNAs, among some of them, are confirmed as possible biomarkers by other studies, e.g., hsa-miR-144-3p, hsa-miR-337-5p, hsa-miR-500a-5p, hsa-miR-26b-5p, hsa-miR-125a-3p, and hsa-miR-19b-3p [44]. Moreover, the expression of miRNAs seems to be different in high-grade serous ovarian carcinoma (HGSC), clear cell ovarian carcinoma (CCC), and ovarian surface epithelium (OSE). miR-509-3-5p and miR-509-5p differentiate CCC from HGSC, while miR-200c-3p is considered to be associated with survival in HGSC patients [45].

Exosomal miR-373, miR-200a, miR-200b, and miR-200c were detected in the serum of EOC patients, and there is a correlation between the increased levels of miR-200b and miR-200c and CA125 values, suggesting that these microRNAs may be involved in tumor progression [37].

## 3. Ovarian Cancer and miRNA Expression Profiles

Multiple miRNAs have been considered as potential clinical cancer biomarkers, particularly in ovarian cancer, and their expression profiles have been analyzed in ovarian cancer cell lines (Table 1), in xenograft mice models for ovarian cancer (Table 2), in surgically excised specimens from ovarian cancer patients (Table 3), or in the serum/plasma of ovarian cancer patients (Table 4).

There is a need for a specificity of at least 99.6%, sensitivity of at least 75%, and a positive predictive value of at least 10% to be considered as effective tools to be able to detect early stages in ovarian cancer patients, especially if one aim to use liquid biopsies [61]. There are few relevant studies for the importance of circulating miRNAs in the early diagnosis of ovarian cancer. The first one was performed by Taylor et al. in 2008, when eight exosomal miRNAs: miR-21, miR-141, miR-200a, miR-200b, miR-200c, miR-203, miR-205, and miR-214 were reported to be elevated in the sera of ovarian cancer patients and identical to microRNAs from ovarian tumor cells, while the same exosomal miRNAs were absent in the normal controls [41]. Taylor and collaborators concluded at that time that these microARNs could be used as surrogate diagnostic markers for biopsy profiling [41].

Over time, in the search for finding a convenient and effective method for early detection of cancer, based on the use of liquid biopsy and with high sensitivity, several other papers were published. As, for example, the one by Resnick et al., which describes a novel real-time PCR microarray detection method [62]. This study provides evidence that eight miRNAs determined in human serum that were obtained from untreated ovarian cancer patients were significantly differentially expressed between the cancer and normal specimens. Among miRNAs-21, 92, 93, 126, and 29a, which were significantly over-expressed in the serum from cancer patients compared to controls, they identified three potential oncomirs: mirs-21, 92, and 93 present in the serum of patients with normal CA-125 [62].

In 2015, Gao et al. identified microRNA-200c and microRNA-141 in the serum of 74 epithelial ovarian cancer patients, and validated the results while using the area under the ROC curve (AUC) and Kaplan–Meier curve and the log-rank test to detect the prognostic value of these microARNs [63].

Zuberi et al. suggested by Trizol method, where miR-200a, miR-200b, and miR-200c overexpressions are associated with the aggressive tumor progression [64]. In addition, Meng et al. found elevated levels of circulating exosomal microRNAs while using TaqMan MicroRNA assays and ELISA. The study was performed in a cohort of 163 epithelial ovarian cancer, and increased levels of exosomal miR-200b and miR-200c mainly observed in advanced EOC patients, while the levels of miR-373 and miR-200a were increased in all FIGO stages [37].

A more recent study developed an optimal detection method after a japanese national project that was entitled Development and Diagnostic Technology for Detection of miRNA in Body Fluids was launched. Among 13 types of cancer, they also included, as an objective, the development of a novel screening strategy capable of discriminating ovarian cancer patients from healthy women. Using 28 cases of ovarian tumors, Yokoi et al. validated their model in an independent cohort and obtained a high accuracy (sensitivity, 0.99; specificity, 1.00) [65].

Several other miRNAs are thought to bring useful information for an early diagnosis of ovarian cancer. The most important data are corroborated in Table 4.

## 4. miRNAs in Ovarian Cancer Invasion and Metastasis

In general, in ovarian cancer, metastasis is promoted by the epithelial-to-mesenchymal transition (EMT), and miRNAs are related to the regulation of signaling proteins that are involved in the control of EMT [68]. Different miRNAs modulate EMT-associated signaling genes. miR-7 is critical in the reversion of EMT due to AKT and ERK1/2 pathways inactivation, by reducing the epidermal growth factor receptor (EGFR) expression and thus inhibiting tumor metastasis [69]. It was suggested by Zhou et al. to be a future therapeutic target for ovarian cancer metastasis intervention [70]. miR-150 is usually downregulated in epithelial ovarian cancer and it is considered as an independent prognostic biomarker that is unique to metastases [71]. miR-150 inhibits cell invasion and metastasis by targeting transcriptional repressor Zinc Finger E-Box Binding Homeobox 1 (ZEB1) and suppressing it [72]. Although miR-146a was suggested to act as a tumor suppressor through the down-regulation of the NFkB activators IRAK1 and TRAF6 [73], another mechanism seems to be responsible for increased survival [71]. The low expression of miR-146a is considered to be of poor prognosis and suggestive of an unfavorable outcome in patients with epithelial ovarian cancer [74]. Some other miRNAs, miR-22, miR-183, and miR-31, have a negative effect on Tiam1 expression and they are responsible for the suppression of cell migration and invasion of serous ovarian carcinoma [75].

miR-17-5p influences ovarian cancer progression, which also influences EMT by targeting PTEN signaling. Experimental studies that were performed on OVCAR-3 and SKOV-3 cell lines showed that miR-17-5p increased migration, while the administration of an anti-miR-17-5p inhibitor decreased the migration and invasion of ovarian cancer cells [76].

Recent studies have demonstrated that exosomes-contained miRNAs have an important contribution to the cell-to-cell communication [13,77]. In particular, this type of intercellular communication that is based on exosomes-contained miRNAs is involved in the metastasis process that is associated with multiple subtypes of cancer [78], including ovarian cancer [79].

## 5. miRNAs and Chemotherapy Sensitivity

Tumor cell resistance to chemotherapeutic agents is a major problem in cancer treatment. Recent studies point to miRNAs as potential therapeutic agents that may play an important role in modulating the tumor cells’ sensitivity to different drugs [80].

Several studies focused on the effects of miRNAs in modulating the ovarian cancer cells’ sensitivity to chemotherapeutic agents (e.g., cisplatin, paclitaxel). Vang et al. demonstrated that miR-150 and miR-146a had distinct effects on cisplatin tolerance in ovarian cancer cells, with miR-150 moderately increasing cisplatin tolerance (i.e., IC50) in SKOV-3 cells, but not in OVCAR-8 and IGROV-1 cells, while miR-146a was ineffective [71]. Zou et al. demonstrated that miR-630 decreased cell proliferation and increased ovarian cancer cells (A2780 and SKOV3) sensitivity to cisplatin, due to its ability to modulate phosphatase and tensin homolog (PTEN) expression in these cells [81]. Additionally, Yang et al. demonstrated that miR-214 increased the ovarian cancer cells (e.g., HIOSE-80, MCC-3, A2780S, and OV119) resistance to cisplatin, by targeting PTEN, which in turn activated the PI3K-Akt pathway and further increased ovarian cancer cell proliferation [82]. Pink et al. have also shown that miR-21-5p increased sensitivity to cisplatin while using ovarian cancer cells (A2780), while miR-21-3p increased their resistance to the drug by targeting neuron navigator 3 (NAV3) gene [83]. Li et al. also demonstrated that miR-137 increased the sensitivity to cisplatin of ovarian cancer cells (SKOV-3 and A2780) and increased the number of apoptotic cancer cells, by regulating the expression of the X-linked inhibitor of apoptosis protein (XIAP) [84]. Additionally, Tian et al. demonstrated that miR-595 overexpression suppressed ovarian cancer cell proliferation, colony formation, and invasion, and that miR-595 sensitized ovarian cancer cells to cisplatin by targeting ATP-binding cassette sub-family B member 1 (ABCB1) [85].

Paclitaxel is another drug used to treat ovarian cancer, to which some tumor cells are resistant. It has been revealed that the inhibition miR-106a and miR-591 expression decreased the level of tumor cells resistance to paclitaxel, as both types of miRNAs are involved in the regulation of pro-apoptotic genes, such as tumor necrosis factor (TNF) ligand/receptor and caspase families. These effects are associated with the capacity of miR-106a and miR-591 to directly target genes that encode for Zinc finger E-box-binding homeobox 1 (ZEB1), B-cell lymphoma/leukemia 10 (BCL 10), and caspase-7 [86]. Additionally, miR-146 increased tumor cell (CAOV3 cells) sensitivity to paclitaxel by downregulating the expression of superoxide dismutase 2 (SOD2) [52], although it was ineffective in regulating the sensitivity to cisplatin [71].

Helleman et al. highlighted the resistance of ovarian cancer cells to chemotherapy drugs (e.g., paclitaxel and cisplatin), due to changes that occur in the extracellular matrix, alterations that lead to resistance to chemotherapy, but also to cancer progression. The authors argued the role of the transforming growth factor-beta (TGF beta) in gene clusters that are associated with the extracellular matrix. miR-200 downregulation targets TGF beta, which in turn stimulates tumor proliferation, cancer cell metastasis [87], and chemotherapy resistance of ovarian tumor cells [88]. One benefit of miRNA in chemotherapy is that the overexpression of miR can induce sensitivity to several drugs at the same time in tumor cells. In ovarian cancer, miR-146b inhibited the expression of F-box and leucine-rich repeat protein 10 (FBXL10) and also upregulated Cyclin D1, vimentin (VIM), and *zona-occludens-1* (ZO-1) [53]. Additionally, Yan et al. demonstrated, in SKOV-3, OVCAR-3, HO8910, and A2780 cells, that the overexpression of miR-146b can induce cisplatin and paclitaxel sensitivity, and also confirmed the results of in vivo experiments on nude mice [53]. To resume, the authors pointed out that the overexpression of the miR-146b slowed the progression of malignancy, while miR-146b downregulation had opposite effects [53].

The low expression of miR-133b [89], miR-186 [90], and miR-873 [91] in A2780 and OVCAR-3 cells was associated with the upregulation of drug-resistance-related genes. Studies aiming to identify their role in ovarian cancer have shown that all of these three miRNAs mediate glutathione S-transferase-π (GST-π) and multidrug resistance protein 1 (MDR1) modulation via targeting ATP-binding cassette targeting sub-family B [MDR/TAP], member 1 (ABCB1) [90,91,92]. Thus, by the overexpression of miR-133b, miR-186, and miR-873, the expression of the genes mentioned above were downregulated in tumor cells, being accompanied by an increased sensitivity of ovarian cancer cells to cisplatin and paclitaxel [89,90,91]. However, by targeting the same genes, other miRNAs have opposite effects. In detail, miR-130b increased resistance in A2780 and A2780/Taxol cells for cisplatin and paclitaxel, by upregulating GST-π, MDR1, and P-glycoprotein (P-gp) [93]. Additionally, increased levels of miR-490-3P in A2780 and A2780/Taxol tumor cells decreased the paclitaxel sensitivity and upregulated GST-π, MDR1 and P-gp [89,94]. Another oncomiR, miR-181a-5p, was described by Petrillo et al. in tumor cells that were isolated from biopsies of patients who received neoadjuvant chemotherapeutic treatment. miR-181a-5p was demonstrated to activate the TGF-beta pathway, resulting in increased cell resistance to platinum treatments [95]. To resume, miRNAs can modulate the activity of several genes that are involved in apoptosis, DNA repair, or drug resistance in tumor cells, so that it may be seen as a possible future approach in cancer treatment [96].

## 6. miRNAs and Their Therapeutic Potential

In conventional chemotherapy treatment, drugs, such as topoisomerase inhibitors, inhibitors of microtubule polymerization, alkylating agents, antimetabolites, platinum agents, and hormonal agents, which have the role of disrupting the cell cycle and inhibiting cell division, are frequently used [97]. Another approach in cancer therapy is the use of miRNAs as a strategy for obtaining safe treatments that are based on small molecules in different types of cancer [98].

The therapeutic potential of miRNAs can be exploited through the overexpression of miRNAs as tumor suppressors, while using synthetic miRNAs mimics [99,100] or molecules that inhibit the activity of oncogenic miRNAs [101]. Some examples of the effects that are induced by miRNAs can be summarized:

- The inhibition of tumor proliferation, by the downregulation of genes and pathways involved in this process. In detail, miR-218 is targeting Runt-related transcription factor 2 (RUNX2) [102], miR-199a overexpression produces a downregulation of CD44 [103], miR-532-5p functions as a tumor suppressor by downregulation of casein kinase II subunit alpha 2 (CSNK2A2), SH3 and PX domain-containing protein 2A (SH3PXD2A), and Chromodomain-helicase-DNA-binding protein 4 (CHD4) [104], miR-222-3p and miR-221 reduce tumor cell proliferation by inhibiting AKT phosphorylation [105] and miR-135a-3p mediates the expression of Baculoviral IAP repeat-containing protein 3 (BIRC3), GAGRA3, and Sperm protein associated with the nucleus, X chromosome (SPANX) B1/SPANX B2 [106];
- Apoptosis of cancer cells, which occurs by miR-29b inducing PTEN hypomethylation (tumor suppressor), and, in turn, increases the expression of PTEN in cancer cells by directly binding to the 3′-UTR of PTEN [107]. miR-491-5p induces tumor cell apoptosis by inhibiting BCL-XL and EGFR signaling [108], and miR-744-5p induces tumor cell apoptosis in ovarian cancer by targeting Heterogeneous nuclear ribonucleoproteins C1/C2 (HNRNPC) and Nuclear factor 1 X-type (NFIX) [109];
- Reduction in the rate of migration and formation of tumor metastases: miR-200 regulates IL-8 and CXCL1 activity [110], miR-199a is targeting Hypoxia-inducible factor (HIF)-1α and HIF-2α [111], miR-92α mediates the suppression of peritoneal metastasis by inhibiting α5 integrin [112] and miR-143-3p while using TGF-beta activated kinase 1 (TAK1) signaling [113];
- It affects tumorigenesis and chemosensitivity by modulating oxidative stress through the miR-141 and miR-200a regulation at the level of p38α protein [114];
- Inhibition of upregulated oncogenic miRs by administration of antisense oligonucleotides and antagomirs or by locked nucleic acid constructs (LNA) [101,115];
- The use of therapeutic approaches based on miRNAs in cancer therapy can have multiple benefits. Among these benefits, the most important is the ease of miRNA administration and delivery. In comparison with other molecules used in gene therapy (e.g., large viral vectors or plasmids), miRNAs can be manipulated easier and delivered due to their low molecular weight and small size [94].

## 7. Molecular Clustering Analysis in Ovarian Cancer

The study of miRNAs facilitates the early clinical prognosis of ovarian cancer due to the recent advances in molecular clustering analysis methods. Advanced statistical analysis allows for the unbiased evaluation of patient cohorts, by pre-processing and analyzing relevant genes expressions.

Clustering methods subsume under the unsupervised learning techniques category and they are used for the classification of data by correlating similar relevant characteristics, with no predefined classes being required [116]. Statistical software packages (i.e., hclust in R) provide fast and optimizable tools for the minimization of inter-class similarity, along with the maximization of intra-class similarity. Studies suggest a non-linear trend in the relationship evaluation among the different species of miRNA; therefore, univariate analysis is often followed by hierarchical clustering [117]. Variability in miRNA expression datasets was evaluated by decomposition of data into isolated patterns, allowing for the application of singular-value decomposition methods. Principal component analysis (PCA) was employed for the identification of significant batch effects among the miRNA expression datasets, and empirical Bayes methods allowed for the adjustment of this effect.

A study by McDonald et al. highlighted the efficiency of variable-based approaches in the clustering of non-responding patients, followed by univariate analysis of biologically relevant variables, among which they evaluated the differential expression of miRNA [118]. miRNA expression patterns were identified and correlated with ovarian cancer samples through PCA [119,120,121], arriving at a highly differentiated profile among ovarian tumoral cell lines (e.g., OVCAR3, OVCAR420 etc), ovarian cancer tissues, and non-tumorigenic cells (HOSE-B). Another approach for determining differential expression between groups is constituted by the *t*-statistic method linear models for microarray data (LIMMA) [122]. The agglomerative hierarchical clustering method, as employed by Dahiya et al., used a complete linkage method to test the natural tumor samples grouping based on gene-expression profiles correlations [119].

Luo et al. introduced CoModule, a new cluster-based method for the identification of miRNA regulatory modules (MRMs). The MRMs are identified by determining the co-expression clusters through rough set clustering, applying least absolute shrinkage and selection operator (LASSO) regression to reconstruct the regulatory pairs of miRNA and mRNA and adding the targeted mRNA into the miRNA cluster [123]. The study also reported the increased efficiency of the LASSO model in achieving the best performance in terms of the specificity and sensitivity for ovarian cancer data.

The Maximum Weighted Merger Method (MWMM) approach facilitated the clustering of miRNAs and mRNAs that are based on their similar underlying causal factors in cancer. The method consists of a combination between the Hungarian algorithm [124] and the blossom algorithm [125] for the passive generation of clusters from a miRNA-mRNA bipartite graph [126]. Thus, the potential biomarkers can be identified to point out the precision targets for chemotherapy.

Neural networks proved to be highly efficient for ovarian cancer prognosis, as stated in a study by Elias et al., in which batch adjustments were performed before the analysis. The neural network expressed a high area under the curve (AUC) value of approximately 0.9, in contrast to other classification models, on both pre-processed and raw data. The model that was employed was a multilayer perceptron with the number of hidden layers being optimized to avoid overfitting [127]. Differences in false-positive and false-negative assignment were compared while using Fisher’s exact test, and by the use of 14 miRNAs from pre-and post-operative original signature, provided very good discriminatory power on the testing set (AUC 0.93, 95% CI 0.81–1.00), with an overall sensitivity of 75% and specificity of 100% [122].

A study by Hu et al. indicated a significant association between the identified miR-200 cluster through expression profiling analysis. Additionally, it has been reported that miR-200b-429 clusters were being co-regulated and targeting multiple genes playing a role in cancer. Therefore, they might be seen as predictive markers in ovarian cancer. In their predictive model, the links between the disease outcome and miRNA were tested while using the Cox Proportional-Hazard model, while the relationship between each miRNA and the outcome prognostic was carried out using the non-parametric Kaplan–Meier estimator [128].

Based on a training set from The Cancer Genome Atlas (TCGA) database, a Support Vector Machine (SVM) classifier, along with a recursive feature elimination (RFE) algorithm was built [129]. The miRNA categories were selected prior to classification by Cox regression analysis and RFE, arriving at an optimum of 19 miRNAs. The favorable AUC values of both training and validation sets (>0.9) suggested a significant relationship between the SVM results and prognosis, therefore indicating that the classifier based on 19 miRNAs could evaluate the recurrence of ovarian cancer [130].

The computational limitations in the molecular cluster analysis of miRNA are increased by the high computational complexity of biological data processing. Therefore, k-means clustering became highly employed in studies due to its low computational complexity [59]. Table 5 contains a detailed comparison between hierarchical and k-means clustering, while pondering on the advantages and disadvantages of miRNA data analysis.

## 8. Conclusions and Future Perspectives

Ovarian cancer is characterized by a very high mortality rate being diagnosed at a late stage, which is mainly due to the lack of early detection tools. This review summarized the role of microRNAs as clinical cancer biomarkers for ovarian cancer. Screening serum/plasma miRNAs might contribute to improved prognosis of ovarian cancer, but first, there is a need to understand whether, how, and when miRNAs might be used as biomarkers. An important distinction should be made between miRNA as clinical biomarkers in the histological exam that is based on the analysis of surgically excised specimens from ovarian cancer patients and miRNAs as clinical biomarkers in serum/plasma of ovarian cancer patients. The miRNA expression profiles in patients’ serum/plasma are more relevant for early diagnosis of ovarian cancer, while the miRNA expression profiles in histological specimens are only useful for intraoperative diagnosis confirmation. The most robust miRNA expression profiles are those that are tested in a variety of samples (e.g., ovarian cancer tissue obtained from surgical resections, plasma/serum from ovarian cancer patients, ovarian cancer cells, xenograft mice, etc.). However, many studies so far have focused on alterations of single microRNA or of a reduced number of selected microRNAs in ovarian cancer. Few studies have considered the validation of miRNA expression profiles by molecular clustering analysis.

In perspective, the choice of the best microRNAs-based assays, which were validated in both extensive clinical screenings and bioinformatics approaches, will enable rapid, efficient, and cost-effective detection of ovarian cancer in early stages. Furthermore, miRNA expression profiles may be affected by pharmacological treatments and, therefore, we can assume that they have the potential to serve as therapeutic targets/agents for the treatment of ovarian cancer.

## Figures and Tables

**Table 1:** miRNA in ovarian cancer cell lines.

miRNA	Type of Ovarian Cells	Changes in Ovarian Cancer
miR-23b	OVCAR3, HO8910-PM, SKOV3/DDP cells	miR-23b transfection slows down cell growth, blocks cell cycle in G1, increases the number of apoptotic cells, and reduces the rate of cell migration [46]
miR-26a	SKOV-3, ES2 cells	Overexpression of miR-26b in ovarian cancer cells [47] miR26-b is involved in tumor progression by targeting estrogen receptor alpha (ERα) [47]
miR-125b	SKOV3 cells	Overexpression or downregulation of miR-125b did not affect the in vivo cancer cells proliferation and apoptosis [48]
miR-125b	SKOV3, ES2 cells	Low expression of miR-125b in ovarian tumor cells Through overexpression of miR-125b, tumor proliferation is prevented by controlling the G2 phase of the cell cycle and by targeting the BCL3 gene [49]
miR-141	Ovarian cancer cells (SKOV3, OVCA433, and A2780cp) Cervical cancer cells (OV2008 and C13*)	miR-141 increases anchorage-independent growth and survival of ovarian cancer cells in vitro [50] miR-141 enhances anoikis resistance in metastatic tumor progression by targeting the Kruppel-related zinc finger protein AP-2rep (KLF12)/specificity protein 1 (Sp1)/survivin axis
miR-145 miR-133b	Ovarian cancer cells (3AO, SKOV3)	miR-145 promotes miR-133b expression through c-myc and DNMT3A-mediated methylation in ovarian cancer cells [51] miR-133b upregulation mediates the inhibitory activity of miR-145, in aerobic glycolysis conditions (i.e., Warburg effect), by targeting hexokinase-2 (HK2) in ovarian cancer cells [51]
miR-146a	Epithelial ovarian cancer cells (OVCAR3, CAOV3, HEY) Normal ovarian cells (HOSE)	Through overexpression, it acts as a tumor suppressor, but through its downregulation, it inhibits apoptosis, increases proliferation and chemoresistance [52]
miR-146b	Epithelial ovarian cancer cells (SKOV3, OVCAR3, HO8910, A2780)	miR-146b overexpression upregulates VIM and ZO-1 and consequently inhibits tumor dissemination [53]
miR-148a	Ovarian cancer cells (SKOV3, OVCAR, and A2780) Normal ovarian epithelial cells (HUM-CELL-0088)	Downregulation of miR-148a in ovarian cancer cells [54] miR-148a inhibits migration and invasion of ovarian cancer cells via targeting sphingosine-1-phosphate receptor 1 (S1PR1) [54]
miR-200a-3p	Ovarian cancer cells (ES2, HO8919PM, SKOV3, HO8910) Ovarian surface epithelial cells (HOSEpiC)	Overexpression of miR-200a-3p strongly promotes the proliferation, colony formation and invasion of ovarian cancer cells [55] Binds the 3′-UTR of PCDH9 and decreased the expression of PCDH9 [55]
miR-337-3p	Ovarian cancer cells (ES2, A2780, SKOV-3, OVCAR-3)	miR-337-3p inhibits cell proliferation and decreases the PI3K/AKT signaling pathway activation (its targets are PIK3CA and PIK3C) [56] Its ectopic expression inhibits proliferation and induces apoptosis and cell cycle arrest in G0/G1 phase [56]
miR-433	Ovarian cancer cells (SKOV3 and OVCAR3)	miR-433 inhibits migration and invasion of ovarian cancer cells via targeting Notch1 [57]
miR-630	Ovarian cancer cells (SKOV3)	miR-630 overexpression stimulates in vitro cell proliferation and migration [58] miR-630 targets directly Krüppel-like factor 6 (KLF6) in ovarian cancer cells [58]
miR-802	Epithelial ovarian cancer cells (OVCAR3, SKOV3, A2780, and CAOV3) Normal ovarian surface epithelial cells (HOSEpiC)	miR-802 is downregulated in epithelial ovarian cancer cell lines [59] Overexpression of miR-802 suppresses migration, proliferation, invasion and induces apoptosis in epithelial ovarian cancer cell lines [59] miR-802 directly targets Monooxygenase/Tryptophan 5-Monooxygenase Activation Protein Zeta (YWHAZ) gene in epithelial ovarian cancer cells [59]
miR-1271	Ovarian cancer cells (SKOV3 and CAOV3) Normal ovarian cells (IOSE80)	Suppresses cell viability and invasion in ovarian cancer cells [60] Directly binds the 3ʹ-UTR of ZEB1 mRNA and regulates the expression of ZEB1 [60]

**Table 2:** miRNA in ovarian cancer tissue—animal model studies.

miRNA	Type of Ovarian Tissue	Changes in Ovarian Cancer
miR-23b	Nude mice injected subcutaneously with mock or hsa-miR-23b–transfected OVCAR3 cells	miR-23b induces downregulation of cyclin G1 (CCNG1) in tumor xenografts and reduction of tumor size in mice [46]
miR-26a	Nude mice injected subcutaneously with SKOV3 cells transfected with miR-26a or anti-miR-26a	miR-26a is involved in cell proliferation and tumor development in epithelial ovarian cancer induced in animal models [47]
miR-125b	Nude mice inoculated with SKOV3 cells that were transfected with the vector control, miR-125b mimic or inhibitor	miR-125b inhibits the in vivo cancer cell migration and invasion [48]
miR-125b	Nude mice injected subcutaneously with SKOV3 cells transfected with miR-125b or anti-miR-125b	miR-125b suppresses the development of ovarian cancer [49]
miR-141	BALB/cAnN nude mice injected intraperitoneal with stable SKOV3 miR-141-expressing clones, or A2780cp shSu *knockdown* clone and the scrambled controls	miR-141 increases tumor growth in vivo and induces the appearance of a great number of macroscopic tumor nodules, especially in the omentum and the peritoneal cavity [50]
miR-146b	Nude mice injected with control cells or cells overexpressing miR-146b	Overexpression of miR-146b reduces cell migration and decreases the level of F-box and leucine-rich repeat protein 10 (FBXL10) protein [53]
miR-337-3p	Xenograft models of ovarian cancer induced by inoculation of A2780 and OVCAR-3 cells in female BALB/c athymic nude mice	miR-337-3p is a tumor suppressor that controls the expression of p110α and p110β (i.e., PIK3CA and PIK3CB encoded proteins) [56] Tumors injected with miR-337–3p agomiR grew more slowly than those injected with agomiR-NC for both xenograft models [56]
miR-630	Balb/c mice injected subcutaneously with SKOV3 cells transfected with inhibitors, mimics or negative control of miR-630	miR-630 overexpression stimulates in vivo ovarian cancer proliferation [58]

**Table 3:** miRNA in ovarian cancer tissue—clinical studies.

miRNA	Type of Ovarian Tissue	Changes in Ovarian Cancer
miR-125b	Surgical resection of tumor tissues and the corresponding adjacent normal tissues in epithelial ovarian cancer patients	miR-125b is downregulated in ovarian cancer [48] miR-125b suppresses ovarian cancer progression via suppression of the epithelial-mesenchymal transition pathway by targeting the SET protein (binds directly to the SET 3′-UTR) [48]
miR-141	Surgical specimens of ovarian cancer and normal ovarian tissues	miR-141 is upregulated in clinical ovarian cancer samples having a ~10-fold higher expression than in normal ovary tissues [50]
miR-133b miR-145	Human normal ovarian tissue samples and ovarian carcinomas (e.g., serous cancer, mucinous, endometrioid cancer, clear cell cancer)	miR-145 and miR-133b were downregulated in endothelial ovarian cancer, their expression being positively correlated [51]
miR-148a	Surgical resection of ovarian cancer tissue and their matched normal adjacent tissues	Downregulation of miR-148a in ovarian cancer tissue [54]
miR-200a-3p	Surgically excised tissue from ovarian cancer patients	miR-200a-3p expression was negatively correlated with the PCDH9 expression in ovarian cancer [55]
miR-337-3p	Surgically excised epithelial ovarian cancer specimens	Downregulation of miR-337-3p in epithelial ovarian cancer tissues and correlated with the pathological grade of patients [56]
miR-433	Surgical resections of ovarian cancer tissues and matched normal ovary tissues	Downregulation of miR-433 expression and upregulation of Notch1 expression in ovarian cancer tissues compared with normal ovarian tissues [57]
miR-630	Surgically excised ovarian cancer and normal ovarian tissue samples	miR-630 is upregulated in ovarian cancer [58]
miRNA-802	Surgical specimens of epithelial ovarian cancer and adjacent normal tissues	Down-regulation in epithelial ovarian cancer specimens [59]
miR-1271	Surgical specimens of ovarian cancer tissues and peritumoral normal tissues	Inverse correlation between miR-1271 expression and ZEB1 in ovarian cancer tissues [60]

**Table 4:** miRNA in serum/plasma of ovarian cancer patients.

miRNA	Serum/Plasma	Changes in Ovarian Cancer
miR-26a	Plasma	Upregulation of miR-26a in human epithelial ovarian cancer [47]
miRNA-21 miRNA-29a miRNA-92 miRNA-93 miRNA-99b miRNA-126 miRNA-127 miRNA-155	Serum	Upregulation of miRNA-21, miRNA-29a, miRNA-92, miRNA-93, miRNA-126 in ovarian cancer [62] Downregulation of miRNA-99, miRNA-127, miRNA-155 in ovarian cancer [62]
miR-145 miR-133b	Serum	Expression of miR-145 and miR-133b is significantly decreased in the serum of patients with ovarian cancer [51]
miR-193a-5p	Serum	Combined detection of miR-193-5p, HE4 and CA125 improves the diagnostic efficacy of epithelium ovarian cancer [66]
miR-19a-3p miR-30a-5p miR-645 miR-150-5p miR-191-5p miR-206 miR-34c-5p miR-548a-3p miR-320a miR-574-3p miR-590-5p miR-106b-5p	Plasma	Downregulation of miR-19a-3p, miR-30a-5p, miR-645, miR-150-5p in ovarian cancer [67] Upregulation of miR-191-5p, miR-206, miR-34c-5p, miR-548a-3p, miR-320a, miR-574-3p, miR-590-5p, miR-106b-5p in ovarian cancer [67]

**Table 5:** Hierarchical clustering vs. k-Means clustering in miRNA data analysis with relevance in ovarian cancer.

Hierarchical Clustering	k-Means Clustering
Can’t handle large miRNA expression data—quadratic complexity	Can handle large miRNA expression datasets—linear complexity
Reproducible as every miRNA expressed is assigned a cluster, and the clustering occurs based on the closeness of previously generated clusters.	Unreproducible clustering due to the prerequisite of a random number of clusters.
Produces more intuitive results in the form of a dendrogram.	Produces less intuitive results if data does not group into hyper spherical clusters.
Poor performance and higher time of execution as the number of generated clusters increases.	Higher time of execution associated with large miRNA expression datasets.
